# Multiple measurement analysis of resting-state fMRI for ADHD classification in adolescent brain from the ABCD study

**DOI:** 10.1038/s41398-023-02309-5

**Published:** 2023-02-06

**Authors:** Zhaobin Wang, Xiaocheng Zhou, Yuanyuan Gui, Manhua Liu, Hui Lu

**Affiliations:** 1 State Key Lab of Microbial Metabolism, Joint International Research Laboratory of Metabolic Developmental Sciences, Department of Bioinformatics and Biostatistics, School of Life Sciences and Biotechnology, Shanghai Jiao Tong University, Shanghai, China; 2 SJTU-Yale Joint Center of Biostatistics and Data Science, National Center for Translational Medicine, Shanghai Jiao Tong University, Shanghai, China; 3 MoE Key Laboratory of Artificial Intelligence, AI Institute, School of Electronic Information and Electrical Engineering, Shanghai Jiao Tong University, Shanghai, China; 4 Shanghai Engineering Research Center for Big Data in Pediatric Precision Medicine, Center for Biomedical Informatics, Shanghai Children’s Hospital, Shanghai, China

**Keywords:** ADHD, Diagnostic markers

## Abstract

Attention deficit hyperactivity disorder (ADHD) is one of the most common psychiatric disorders in school-aged children. Its accurate diagnosis looks after patients’ interests well with effective treatment, which is important to them and their family. Resting-state functional magnetic resonance imaging (rsfMRI) has been widely used to characterize the abnormal brain function by computing the voxel-wise measures and Pearson’s correlation (PC)-based functional connectivity (FC) for ADHD diagnosis. However, exploring the powerful measures of rsfMRI to improve ADHD diagnosis remains a particular challenge. To this end, this paper proposes an automated ADHD classification framework by fusion of multiple measures of rsfMRI in adolescent brain. First, we extract the voxel-wise measures and ROI-wise time series from the brain regions of rsfMRI after preprocessing. Then, to extract the multiple functional connectivities, we compute the PC-derived FCs including the topographical information-based high-order FC (tHOFC) and dynamics-based high-order FC (dHOFC), the sparse representation (SR)-derived FCs including the group SR (GSR), the strength and similarity guided GSR (SSGSR), and sparse low-rank (SLR). Finally, these measures are combined with multiple kernel learning (MKL) model for ADHD classification. The proposed method is applied to the Adolescent Brain and Cognitive Development (ABCD) dataset. The results show that the FCs of dHOFC and SLR perform better than the others. Fusing multiple measures achieves the best classification performance (AUC = 0.740, accuracy = 0.6916), superior to those from the single measure and the previous studies. We have identified the most discriminative FCs and brain regions for ADHD diagnosis, which are consistent with those of published literature.

## Introduction

Attention deficit hyperactivity disorder (ADHD), typically characterized by the symptoms of inattention, hyperactivity, and impulsivity, has become one of the most common functional disorders in children and adolescents [1]. For example, 8.4% of children from 2 to 17 years of age in the United States were undergoing ADHD, representing 5.4 million children [2]. To date, behavior-based evaluations are the standard clinical approach to diagnosing ADHD, but it is time-consuming and subjective. Moreover, little is known about the association between brain biomarkers (such as brain functional connectivity) and ADHD diagnosis. To tackle this challenge, researchers tend to integrate machine learning (ML) models and brain magnetic resonance imaging (MRI) data for automatical diagnosis and aberrant neuroimaging biomarker identification [3, 4].

Functional magnetic resonance imaging (fMRI), due to its non-invasive and high-resolution properties, has emerged as one of the most frequently used approaches to measuring brain functional connectivities and studying psychiatric diseases [3–6]. Specifically, fMRI detects the changes of deoxyhemoglobin concentration in local blood vessels of the brain during certain tasks or while remaining still [7]. Accordingly, resting-state fMRI (rsfMRI) can reveal ongoing neural and metabolic activities without an explicit task, facilitating studying of functional regions and networks of the brain and temporal associations among them.

In the past few years, rsfMRI has been extensively utilized to diagnose ADHD and discover the brain’s functional differences between ADHD and healthy controls. Voxel-wise and region of interest (ROI)-wise quantitative features extracted from fMRI can reflect local brain activity and brain region connectivity respectively, therefore they have the potential to serve as biomarkers of ADHD and aid clinical assessment. The voxel-wise measures, such as the regional homogeneity (ReHo), amplitude of low-frequency fluctuations (ALFF), and fractional ALFF (fALFF), are calculated at the voxel level. ReHo [8] measures the similarity or synchronization of the time series in a spatial cluster (usually 27 neighboring voxels), while ALFF [9] focuses on the amplitude of regional activity within the frequency range between 0.01 and 0.1 Hz. FALFF [10] is a normalized ALFF, defined as the original ALFF divided by the total power in the detectable frequency range. On the other hand, in ROI-wise analysis, the whole brain will firstly be parcellated into multiple ROIs according to the brain anatomical or functional atlas [11–19]. Then FC matrices that indicate correlative and causal relationships between these predefined ROIs, including the low-order FC (LOFC) and high-order FC (HOFC) networks can be measured. The most popular LOFC construction method is PC analysis, which can capture the pairwise temporal synchronization between two ROIs. However, the limitation of revealing low-order correlation between two brain regions renders the PC-based method notoriously unsuited to capturing high-level relationships among the brain regions. Accordingly, HOFCs, such as tHOFC [20] and dHOFC [21], were proposed to handle this limitation and characterize higher-level brain functional interaction. Besides PC analysis, another category of widely used FC estimation that conducts the partial correlation is the SR method [22], which can characterize the multi-ROI relationship. Moreover, by integrating biological constraints into SR, the generated group SR (GSR) [23], sparse low-rank (SLR) [24], weighted SR (WSR) [25], strength-weighted sparse group representation (WSGR) [25, 26] and strength and similarity guided GSR (SSGSR) [27] are more meaningful for mental disease diagnosis.

In the existing studies on computer-aided ADHD diagnosis, ALFF [9, 28–30], ReHo [31–33], fALFF [34–36], PC-derived FC [37–40], and SR-derived FC [41, 42] mentioned above were widely adopted as potential predictors. However, the high-order FC and optimized SR, encoding more biologically meaningful clues, were rarely utilized in the automatic diagnosis/classification of ADHD. These measures have shown the potential to improve disease diagnosis performance [43–45]. In addition, current studies with high accuracy usually were demonstrated on samples of small size and mostly from a single site [32, 33, 38]. Classification accuracy decreases with the increase of sample size, especially for multi-site heterogeneous datasets [3, 4]. Notably, good performance on small and homogeneous samples does not assure generalizability. Accordingly, multi-site datasets like ADHD-200 [46] and ABCD [47] enable the assessment of the established models’ generalizability on the unseen samples.

**Fig. 1:**
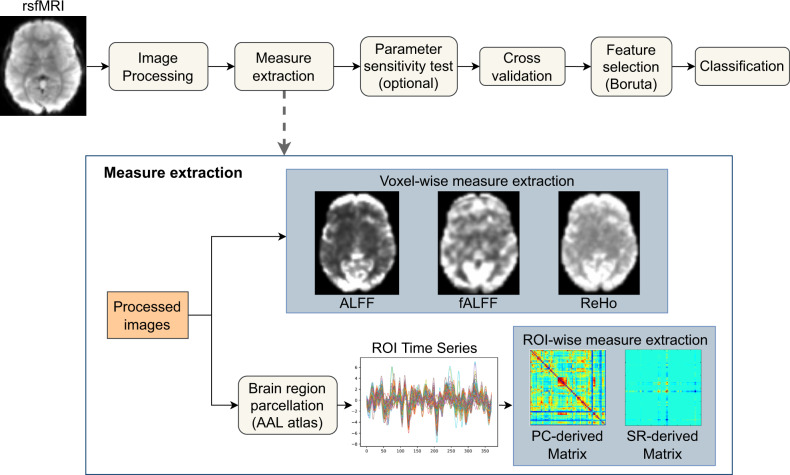
The pipeline of the whole study design. ALFF amplitude of low-frequency fluctuations, fALFF fractional ALFF, ReHo regional homogeneity, AAL automated anatomical labeling, ROI regions of interest, PC Pearson’s correlation, SR sparse representation.

Our study’s experiments have been demonstrated on the multi-site ABCD dataset, including subjects in the 9–10 age range, a narrow one. We extracted ten voxel- and ROI-wise quantitative measures from rsfMRI, and conducted classification using nine basic classifiers combined with Boruta (a Random Forest-based feature extraction method) [48] to diagnose children with ADHD automatically. Nested cross-validation (CV) (10-fold with 5-fold nested) was applied to evaluate the classification of the models. Finally, the model-agnostic and model-based multi-modal fusion approaches were adopted to improve the classification performance by combining the significantly discriminative features. The aims of this study are: (1) to compare the classification performance of different classifiers on different features and (2) to identify biomarkers (brain regions and pairwise connectivity) containing discriminative power of the classification of ADHD. We propose that classification models on HOFCs and optimized SR will achieve better classification performance than traditional measures, and the identified neuroimaging biomarkers will provide new insights into the underlying initial pathogenesis, potentially make ADHD diagnosis and treatment as early as possible. Figure 1 shows the framework of the whole study design. Table 1 shows the abbreviations used in this manuscript.

**Table 1:** Abbreviations used in the manuscript.

Abbreviation	Full term
AAL	Automated anatomical labeling
ABCD	Adolescent Brain and Cognitive Development
AdaBoost	Adaptive Boosting
ADHD	Attention deficit hyperactivity disorder
ALFF	Amplitude of low-frequency fluctuations
ASD	Autism spectrum disorder
AUC	Area under curve
CN	Cerebellum network
CSF	Cerebrospinal fluid
CV	Cross-validation
DAN	Dorsal attention network
dFNC	dynamic functional network connectivity
dHOFC	dynamics-based HOFC
DMN	Default mode network
DPABI	Data Processing & Analysis of Brain Imaging
DPARSF	Data Processing Assistant for Resting-State fMRI
DTI	Diffusion tensor image
FA	Flip angle
fALFF	fractional ALFF
FC	Functional connectivity
FDR	False discovery rate
fMRI	functional magnetic resonance imaging
FOV	Field of view
FPN	Frontoparietal network
FWHM	Full width half maximum
GM	Gray matter
GRF	Gaussian random field
GSR	Group SR
HOFC	High-order FC
KNN	K-Nearest neighbors
KSADS	Kiddie Schedule for Affective Disorders and Schizophrenia
LGBM	Light gradient boosting model
LN	Limbic network
LOFC	Low-order FC
LR	Logistic regression
MCI	Mild cognitive impairment
MKL	Multiple kernel learning
ML	Machine learning
MRI	Magnetic resonance imaging
PC	Pearson’s correlation
ReHo	Regional homogeneity
RF	Random forest
ROC	Receiver operating characteristic
ROI	Region of interest
rsfMRI	resting-state fMRI
SLR	Sparse low-rank
SMN	Sensory-motor network
sMRI	structural magnetic resonance imaging
SPM	Statistical Parametric Mapping
SR	Sparse representation
SSGSR	Strength and similarity guided GSR
SVM	Support vector machine
T1w	T1-weighted
TBI	Traumatic brain injury
TE	Echo time
tHOFC	topographical information-based HOFC
TI	Inversion time
TR	Repetition time
VAN	Ventral attention network
VN	Visual network
WM	White matter
WSGR	Strength-weighted sparse group representation
WSR	Weighted sparse representation

## Materials and methods

### Participants

The data in this study were acquired by the ABCD Research Consortium, which recruited 11,875 children aged between 9 and 10 from 21 research sites across the United States. The project was designed to track their biological and behavioral development from adolescence to early adulthood [47]. Therefore, the subjects would have been followed up for at least ten years. Standardized and harmonized assessments have been established of physical and mental health, neurocognition, substance use, culture, environment, multi-modal structural and functional brain imaging, and bioassay protocols. These assessments would be conducted biennially (imaging and bioassays) or annually (non-imaging) [49]. We used the minimally processed baseline neuroimages and the tabulated brain features processed officially from ABCD Fix Release 2.0.1. ADHD patients were diagnosed according to the ABCD Parent Diagnostic Interview for DSM-5 Full (KSADS-5) of the baseline year [50]. Children with the current ADHD diagnoses were labeled as cases and those without any mental disease diagnosis or any ADHD symptom were as healthy controls. The inclusion criteria were as follows: (1) meeting the recommended MRI inclusion criteria according to the ABCD Fix Note 2.0.1; (2) neither hydrocephalus nor herniation; (3) right-handedness; (4) with improbable or possible mild Traumatic brain injury (TBI); (5) with no missing values in the covariables (sex, age, manufacturer, site information, etc.). According to the officially released issue, we excluded the participants scanned on Phillips devices due to incorrect post-processing. Finally, we preprocessed T1w and fMRI data (detailed in 2.2) and removed the participants without passing QC for normalization and head motion (<0.2 mm). The demographic information of the remaining (775 participants) is listed in Table 2. Supplemental Table 1 shows the number of participants remaining after each exclusion step.

**Table 2:** Demographic information of the dataset used in this study.

Demographic information	ADHD (*N* = 373, 48%)	Control (*N* = 402, 52%)	*P*-value
Age (mean ± SD, months)	118.85 ± 7.37	119.06 ± 7.40	0.683
Gender (female/male, %)	35/65	56/44	0.005
Manufacture (SIEMENS/GE, %)	74/26	88/12	0.019
Handedness (right/mixed, %)	83/17	95/5	0.013

Statistics: Two-sample t-test after Levene’s test (*p*-value = 0.999) for age; Chi-square test for gender, manufacture, and handedness.

### fMRI acquisition and preprocessing

The T1w and functional MRI data were collected on the scanners of two vendors (Siemens and GE scanners), across which a harmonized MRI acquisition protocol with comparable acquisition parameters was established. The T1w images were acquired with the following parameters: matrix of 256 × 256, 176 slices (Siemens) and 208 slices (GE), field of view (FOV) size = 256 × 256, voxel size = 1.0 mm^3^, repetition time (TR) = 2500 ms, echo time (TE) = 2.88 ms (Siemens) and 2 ms (GE), inversion time (TI) = 1060 ms, flip angle (FA) = 8°, and acquisition time 7’12” (Siemens) and 6’09” (GE). The fMRI data were acquired with: matrix of 90 × 90, 60 slices, FOV size = 216 × 216, voxel size = 2.4 × 2.4 × 2.4 mm^3^, TR = 800 ms, TE = 30 ms, and FA = 52°. All MRI data have been run through standard modality-specific preprocessing stages, including raw file compression, distortion correction, movement correction, alignment to standard space, initial quality control, etc. More details about the MRI acquisition, scanning parameters, and preprocessing pipelines are reported in prior work [51].

### Extraction of fMRI measures

The minimally preprocessed fMRI data were further processed using the Data Processing Assistant for Resting-State fMRI (DPARSF v5.1) software (http://rfmri.org/DPARSF), which is based on the toolbox for Data Processing & Analysis of Brain Imaging (DPABI, http://rfmri.org/DPABI) and Statistical Parametric Mapping (SPM, http://www.fil.ion.ucl.ac.uk/spm) [52]. The first ten frames were removed to make data reach equilibrium. The time series of images for each subject were realigned and averaged to a mean volume. Then individual structural images (T1w) were co-registered to the mean fMRI and segmented into gray matter (GM), white matter (WM), and cerebrospinal fluid (CSF). After resampling to 3 × 3 × 3 mm^3^ of voxel size, fMRI images were transformed from individual native space to the Montreal Neurological Institute (MNI) space. Nuisance signals were removed by a linear regression model with head motion parameters, linear trends, and WM and CSF signals included as regressors. Finally, images were spatially smoothed with a 4 mm full width half maximum (FWHM) Gaussian kernel (except for ReHo) and temporally filtered to preserve the signals of 0.01–0.1 Hz (except for fALFF) and remove the high-frequency physiological noise.

We calculated the traditional voxel-wise ALFF, fALFF, and ReHo with DPARSF v5.1. For ROI-wise measures, we first extracted the average time series within each ROI based on the automated anatomical labeling (AAL) template [12] (detailed in Supplementary Table 3). Then the PC-derived LOFC and HOFC (tHOFC and dHOFC), as well as SR-derived FC (SR, GSR, SLR, and SSGSR), were measured with BrainNetClass toolbox v1.1 [45]. After that, the effects of main covariates, including sex, manufacturer, site, and head motion, were regressed, and the residuals were used as inputs for the classification models.

As for PC-derived HOFC, tHOFC can be calculated using the following equation [21, 45]:

1 $$\begin{array}{ll} \begin{array}{lll} t{\rm{HOFC}}_{ij} &= &{\rm{Corr}}\left( {{{{\boldsymbol{FC}}}}_{{{\boldsymbol{i}}}},{{{\boldsymbol{FC}}}}_{{{\boldsymbol{j}}}}} \right)\cr \\ & = &\frac{{\mathop {\sum}\nolimits_{k = 1}^N {\left( {w_{ik} - \overline {w_{i \cdot }} } \right)\left( {w_{jk} - \overline {w_{j \cdot }} } \right)} }}{{\sqrt {\mathop {\sum}\nolimits_{k = 1}^N {\left( {w_{ik} - \overline {w_{i \cdot }} } \right)^2} } \sqrt {\mathop {\sum}\nolimits_{k = 1}^N {\left( {w_{jk} - \overline {w_{j \cdot }} } \right)^2} } }}\end{array} \end{array}$$

where *w*_*ik*_ is the PC between *i*th and *k*th ROI signals, and $i,j,k \in \{ 1,2, \ldots ,N\} ,k\, \ne\, i,j$, *N* is the number of ROIs (in our case, *N* = 116). Note that tHOFC indicates the similarity of the LOFC topographical profiles by measuring the correlation of correlation rather than that of the time series, which reflects the high-level property of the brain network and makes providing supplementary information to the traditional LOFC promising. The calculation of dHOFC is defined as [21, 45]:

2 $$\begin{array}{ll} \begin{array}{lll}dHOFC_{ij,pq} &= &Corr\left( {{{{\boldsymbol{FC}}}}_{{{{\boldsymbol{ij}}}}},{{{\boldsymbol{FC}}}}_{{{{\boldsymbol{pq}}}}}} \right)\cr \\&= &\frac{{\mathop {\sum}\nolimits_{\theta = 1}^{{{\mathrm{{\Theta}}}}} {\left( {w_{ij}\left( \theta \right) - \overline {w_{ij}\left( \cdot \right)} } \right)} \left( {w_{pq}\left( \theta \right) - \overline {w_{pq}\left( \cdot \right)} } \right)}}{{\sqrt {\mathop {\sum}\nolimits_{\theta = 1}^{{{\mathrm{{\Theta}}}}} {\left( {w_{ij}\left( \theta \right) - \overline {w_{ij}\left( \cdot \right)} } \right)^2} } \sqrt {\mathop {\sum}\nolimits_{\theta = 1}^{{{\mathrm{{\Theta}}}}} {\left( {w_{pq}\left( \theta \right) - \overline {w_{pq}\left( \cdot \right)} } \right)^2} } }} \end{array} \end{array}$$

where Θ is the number of sliding windows which depends on whole time length (T), step size (s) and window length (L) (${{{\mathrm{{\Theta}}}}} = \left\lfloor {(T - L)/s} \right\rfloor + 1$, in our case, *T* = 370, *s* = 1), *w*_*ij*_ (*θ*) means PC between *i*th and *j*th ROIs in the *θ*th sliding window. By calculating the correlation between two PC time series, *dHOFC*_*ij*,*pq*_ shows how the PC between the *i*th and the *i*^th^ ROIs influence the PC between the *p*th and the *q*th ROIs. In this way, the total number of elements of dHOFC is proportional to *N*^2^ × *N*^2^, much larger than that of PC and tHOFC (*N* × *N*). Ward’s linkage clustering [53], a widely used hierarchical clustering algorithm, can be applied to group the resulting FCs into K clusters to reduce the dimension of the large-scale network. Each cluster contains several FCs showing a similar pattern of variation along the time. Finally, the averaged correlation time series in each cluster can be calculated to construct a *K* × *K* HOFC network. Thus, the final low-scale dHOFC characterizes the temporal synchronization of dynamic FC time series.

As for the SR method, a sparse FC matrix can be generated by minimizing the loss function:

3 $$\begin{array}{*{20}{c}} {\mathop {{\min }}\limits_{{{\boldsymbol{W}}}} \frac{1}{2}\left\| {{{{\boldsymbol{X}}}} - {{{\boldsymbol{XW}}}}} \right\|_F^2 + \lambda \left\| {{{\boldsymbol{W}}}} \right\|_1} \end{array}$$

where ***X*** is the original fMRI data matrix, ***W*** is the FC matrix, *λ* > 0 controls network sparsity with an l_1_-norm penalty. Another SR-based approach named SLR is formulated as the following [24, 45].

4 $$\begin{array}{*{20}{c}} {\mathop {{\min }}\limits_{{{\boldsymbol{W}}}} \frac{1}{2}\left\| {{{{\boldsymbol{X}}}} - {{{\boldsymbol{XW}}}}} \right\|_F^2 + \lambda _1\left\| {{{\boldsymbol{W}}}} \right\|_1 + \lambda _2\left\| {{{\boldsymbol{W}}}} \right\|_ \ast } \end{array}$$

where *λ*_1_ controls sparsity with an l_1_-norm and *λ*_2_ controls modularity with a trace norm. Thus, SLR resulting FC matrices are sparse and low-rank, i.e., modularized and more biologically meaningful [54]. Formula (5) shows the loss function of GSR [23, 45]:

5 $$\begin{array}{*{20}{c}} {\mathop {{\min }}\limits_{{{{\boldsymbol{W}}}}_{{{\boldsymbol{i}}}}} \mathop {\sum}\limits_{s = 1}^S {\left( {\frac{1}{2}\left\| {{{{\boldsymbol{x}}}}_{{{\boldsymbol{i}}}}^{{{\boldsymbol{s}}}} - {{{\mathbf{{{{\mathcal{X}}}}}}}}_{{{\boldsymbol{i}}}}^{{{\boldsymbol{s}}}}{{{\mathcal{w}}}}_{{{\boldsymbol{i}}}}^{{{\boldsymbol{s}}}}} \right\|_2^2} \right) + \lambda \left\| {{{{\mathbf{{{{\mathcal{W}}}}}}}}_{{{\boldsymbol{i}}}}} \right\|_{2,1}} } \end{array}$$

where ${{{\boldsymbol{x}}}}_{{{\boldsymbol{i}}}}^{{{\boldsymbol{s}}}}$ is the regional mean time series of the *i*th ROI for the *s*th subject, and ${{{\boldsymbol{x}}}}_{{{\boldsymbol{i}}}}^{{{\boldsymbol{s}}}}$ can be regarded as a linear combination of time series of other ROIs: ${{{\boldsymbol{x}}}}_{{{\boldsymbol{i}}}}^{{{\boldsymbol{s}}}} = {{{\mathbf{{{{\mathcal{X}}}}}}}}_{{{\boldsymbol{i}}}}^{{{\boldsymbol{s}}}}{{{\mathcal{w}}}}_{{{\boldsymbol{i}}}}^{{{\boldsymbol{s}}}} + {{{\boldsymbol{e}}}}_{{{\boldsymbol{i}}}}^{{{\boldsymbol{s}}}}$, ${{{\mathbf{{{{\mathcal{X}}}}}}}}_{{{\boldsymbol{i}}}}^{{{\boldsymbol{s}}}} = [{{{\boldsymbol{x}}}}_1^{{{\boldsymbol{s}}}}, \ldots ,{{{\boldsymbol{x}}}}_{{{{\boldsymbol{i}}}} - 1}^{{{\boldsymbol{s}}}},{{{\boldsymbol{x}}}}_{{{{\boldsymbol{i}}}} + 1}^{{{\boldsymbol{s}}}}, \ldots ,{{{\boldsymbol{x}}}}_{{{\boldsymbol{N}}}}^{{{\boldsymbol{s}}}}]$ is data matrix of all time series except for the *i*th ROI, ${{{\mathcal{w}}}}_{{{\boldsymbol{i}}}}^{{{\boldsymbol{s}}}}$ is the weight vector quantifying the degree of influence of other ROIs on the *i*th ROI, thus the dimension of ${{{\mathcal{w}}}}_{{{\boldsymbol{i}}}}^{{{\boldsymbol{s}}}}$ is (*N* − 1), ${{{\boldsymbol{e}}}}_{{{\boldsymbol{i}}}}^{{{\boldsymbol{s}}}}$ is the error, ${{{\mathbf{{{{\mathcal{W}}}}}}}}_{{{\boldsymbol{i}}}} = \left[ {{{{\mathcal{w}}}}_{{{\boldsymbol{i}}}}^1,{{{\mathcal{w}}}}_{{{\boldsymbol{i}}}}^2, \ldots ,{{{\mathcal{w}}}}_{{{\boldsymbol{i}}}}^{{{\boldsymbol{S}}}}} \right]$, *S* is the number of subjects. Controlled by an l_2,1_-penalty, GSR resultant FC matrices have less inter-subject variability than SR. SSGSR, as an optimized GSR, is to minimize the following loss function [27]:

6 $$\begin{array}{*{20}{c}} {\mathop {{\min }}\limits_{{{{\boldsymbol{W}}}}_{{{\boldsymbol{i}}}}} \mathop {\sum}\limits_{s = 1}^S {\left( {\frac{1}{2}\left\| {{{{\boldsymbol{x}}}}_{{{\boldsymbol{i}}}}^{{{\boldsymbol{s}}}} - {{{\mathbf{{{{\mathcal{X}}}}}}}}_{{{\boldsymbol{i}}}}^{{{\boldsymbol{s}}}}{{{\mathcal{w}}}}_{{{\boldsymbol{i}}}}^{{{\boldsymbol{s}}}}} \right\|_2^2} \right) + \lambda _1\left\| {{{{\boldsymbol{B}}}}_{{{\boldsymbol{i}}}} \odot {{{\mathbf{{{{\mathcal{W}}}}}}}}_{{{\boldsymbol{i}}}}} \right\|_{2,1} + \lambda _2\mathop {\sum}\limits_{p,q = 1}^S {l_i^{p,q}\left\| {{{{\mathcal{w}}}}_{{{\boldsymbol{i}}}}^{{{\boldsymbol{p}}}} - {{{\mathcal{w}}}}_{{{\boldsymbol{i}}}}^{{{\boldsymbol{q}}}}} \right\|_2^2} } } \end{array}$$

where ${{{\boldsymbol{B}}}}_{{{\boldsymbol{i}}}} = \left[ {{{{\boldsymbol{b}}}}_{{{\boldsymbol{i}}}}^1,{{{\boldsymbol{b}}}}_{{{\boldsymbol{i}}}}^2, \ldots ,{{{\boldsymbol{b}}}}_{{{\boldsymbol{i}}}}^{{{\boldsymbol{S}}}}} \right]$, ${{{\boldsymbol{b}}}}_{{{\boldsymbol{i}}}}^{{{\boldsymbol{s}}}} = [b_{i,1}^s, \ldots ,b_{i,i - 1}^s,b_{i,i + 1}^s, \ldots ,b_{i,N}^s]$, and $b_{i,j} = e^{ - w0_{i,j}^2}$, *w*0_*i,j*_ is the LOFC between the *i*th and the *j*th ROIs, hence *B*_*i*_ is to penalize the links with weak LOFC. $l_i^{p,q} = e^{ - \left\| {w0_i^p - w0_i^q} \right\|_2^2}$ defines the similarity between the *p*th and the *q*th subjects in terms of their one-to-all LOFC patterns for the *i*th ROI. Therefore, *λ*_1_ and *λ*_2_ can control the weighted sparsity and inter-subject variability separately.

The aforementioned HOFCs and SR methods were proposed and applied to various mental diseases, including mild cognitive impairment (MCI) [24, 27, 43] and autism spectrum disorder (ASD) [44], etc. Concerning ADHD, a study [55] built a diagnostic model based on the temporal variability of dynamic functional connectivity, but it did not depend on sliding windows. Another study [56] utilized dynamic functional network connectivity (dFNC) to access FC differences among child, adolescent, and adult ADHD patients rather than between patients and healthy controls. Moreover, the dFNC analysis process is different from dHOFC. To our knowledge, no other study applies tHOFC, dHOFC, and three SR-derived FC (GSR, SSGSR, and SLR) to the classification of ADHD. For the first time, we extracted these features combined with traditional voxel-wise measures and ROI-wise PC and SR to perform a classification task on ADHD and compare the discriminative power of different measures.

### ADHD classification

We tested the performance of nine classification algorithms, including four basic classifiers (Logistic Regression (LR), K-Nearest Neighbors (KNN), Ridge Classifier, and Gaussian Naïve Bayes (GaussianNB)), two SVM-based classifiers (Linear SVM and non-linear SVM), and three tree-based ensemble classifiers (Random Forest (RF), Light Gradient Boosting Model (LGBM) and Adaptive Boosting (AdaBoost)). Owing to the high dimension and redundancy of the candidate features, we used Boruta [48] to identify the features with discriminative power on the training set. This RF-based feature selection method has been proven effective in our previous study [40]. Noting that indices including dHOFC and SR-derived FCs are based on parameter-required methods, we tested the parameter sensitivity for these indices and chose the suggested parameters for the subsequent nested CV. Specifically, for each parameter (or parameter combination) 10-fold was evaluated, which created a plot of parameter sensitivity showing the changes in the average AUCs corresponding to various parameters. Then the parameter with the highest average AUC was selected. In each CV loop, a non-linear SVM with the default configuration (kernel = ’rbf’ and C = 1.0) was used as the classifier. The details of parameters associated with different indices are shown in Table 3. Finally, we applied two multi-modal classification methods (model-agnostic early fusion strategy and model-based Multiple Kernel Learning (MKL) algorithm [57, 58]) to fusing the discriminative features from different modalities and improving the classification performance.

**Table 3:** Parameter combinations and optimal parameters for different measures.

Measure	Parameter	Values	Suggested value
dHOFC	K	[100, 200, …, 600]	600
	L	[20, 30, …, 70]	50
SR	*λ*	[0.01, 0.02,…,0.1]	0.01
GSR	*λ*	[0.01, 0.02,…,0.1]	0.01
SSGSR	*λ*_1_	[0.01, 0.02,…,0.1]	0.06
	*λ*_2_	[0.01, 0.02,…,0.1]	0.01
SLR	*λ*_1_	[0.01, 0.02,…,0.1]	0.05
	*λ*_2_	[0.01, 0.02,…,0.1]	0.08

K: Cluster Number; L: Window Length.

### Nested cross-validation

**Fig. 2:**
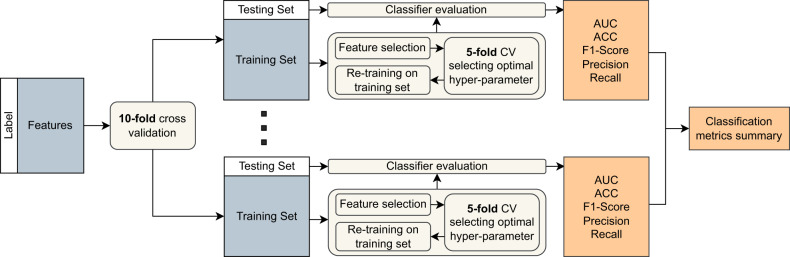
The nested cross validation (CV). The inner CV was to tune the hyperparameters, and the outer CV was to select the discriminative features and evaluate the performance of models.

We applied a nested CV, including an outer 10-fold CV and an inner 5-fold CV, to evaluating the performance of the classification models (shown in Fig. 2). Compared to single CV, the nested CV will get a more accurate estimate of the models’ generalization performance. In the outer 10-fold CV, we applied feature selection on the training set. Then parameter optimization through grid search was conducted on the inner 5-fold CV within the training set. The final model that leads to the highest AUC was re-trained on the training set and evaluated on the testing set of outer CV. This can lead to a more realistic estimate of the models’ generalization performance, since the models have not been overfitted to the test set. Finally, ten folds metrics, including AUC, ACC, F1-score, precision and recall, were summarized as the performance of the models.

The classification metrics can be calculated as follows:

$${\rm{ACC}} = \frac{{TP + TN}}{{TP + TN + FN + FP}}$$

$${\rm{precision}} = \frac{{TP}}{{TP + FP}}$$

$${\rm{recall}} = \frac{{TP}}{{TP + FN}}$$

$$F1\,{\rm{score}} = \frac{{2 \cdot {\rm{precision}} \cdot {\rm{recall}}}}{{{\rm{precision}} + {\rm{recall}}}}$$

where *TP* and *TN* are the counts of correctly classified ADHD patients and healthy subjects respectively, while *FN* and *FP* are counts of falsely classified ADHD patients and healthy subjects respectively. In addition, classification AUC represents the area under the receiver operating characteristic (ROC) curve drawn when the discrimination cutoff varies.

### Biomarker identification

We identified fMRI biomarkers with significant discriminative power in classifying ADHD and the control. For ROI-wise measures, the discriminative features always selected in each outer CV fold were firstly regarded as feature candidates. Then, for each candidate, a two-sample t-test with false discovery rate (FDR) correction was performed between the groups of subjects, and the features with a statistically significant difference were selected as the final biomarkers. However, for voxel-wise measures, the voxels selected across all folds were spatially discontinuous. In other words, they were unable to form clusters. Accordingly, we applied a two-sample t-test with Gaussian random field (GRF) correction to the whole subjects using DPABI. Voxel level value were set *P* < 0.001 and cluster level *P* < 0.05 (two-tailed). The features passing GRF correction were regarded as the final biomarkers.

## Results

### Parameter sensitivity test

**Fig. 3:**
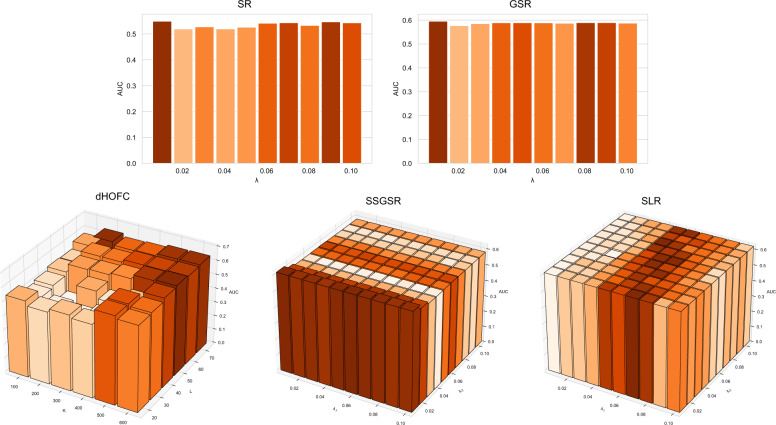
AUC changing with different parameter combinations. Results of SR and GSR with a single parameter are shown in the 2-D bar plot, while dHOFC, SSGSR, and SLR with two parameters are shown in the 3-D bar plot.

We first conducted a parameter sensitivity test on the parameter-dependent measures (PC-derived dHOFC, SR-derived SR, GSR, SSGSR, and SLR) to determine the best parameter (or parameter combination) for the subsequent classification. As shown in Fig. 3, classification AUC is sensitive to parameter combinations for dHOFC, while, for SR-derived measures, AUC changes only slightly with a different parameter (or parameter combination). We chose the parameters with the highest average AUC on the 10-fold CV as the optimal, whose details are shown in Table 3.

### Classification performance

**Fig. 4:**
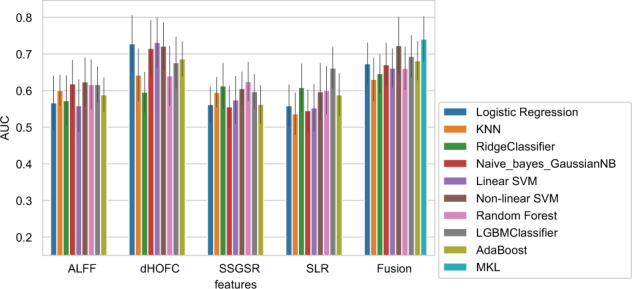
Classification AUC of different measures and multi-modal fusion strategies. Compared to other measures, PC-derived dHOFC shows higher AUCs on the algorithms except for the Ridge Classifier. As for multi-modal fusion, MKL outperformed the early fusion strategy and achieved the highest AUC (0.7408), while both dwarfed the unimodal methods.

The classification performance of nine unimodal classifiers and two multi-modal fusion methods based on four measures of ALFF, dHOFC, SSGSR, and SLR is summarized in Table 4 and Fig. 4. In voxel-wise measures, ALFF outperformed the others with the best AUC (0.624) achieved on non-linear SVM and the best ACC (0.5921) on Gaussian Naïve Bayes. Likewise, dHOFC has the best AUC (0.7315) and ACC (0.675) achieved by linear SVM, performing better than the other ROI-wise PC-derived measures. In SR-derived measures, SSGSR and SLR had better performance than the others: SLR achieved the best AUC (0.6616) and ACC (0.6219) by LGBM, SSGSR the best precision (0.7157) by Ridge Classifier. Furthermore, MKL reached the highest AUC (0.7408) and ACC (0.6916) and outperformed all unimodal methods, which can be explained by the combination of complementary neuroimaging information reflected by different measures. The full results based on the ten measures can be found in Supplemental Table 2.

**Table 4:** Classification performance of different measures and multi-modal fusion methods.

Feature type	Classifier	AUC	ACC	F1-score	Precision	Recall
**ALFF**	Logistic regression	0.5668	0.5446	0.5157	0.529	0.5063
	KNN	0.6007	0.5613	0.3543	0.6043	0.2574
	RidgeClassifier	0.5724	0.5536	0.5159	0.5388	0.4983
	Naïve_bayes_GaussianNB	0.6183	**0.5921**	0.4746	**0.627**	0.3888
	Linear SVM	0.5588	0.5446	0.515	0.5272	0.5066
	Non-linear SVM	**0.624**	0.5819	**0.5599**	0.5662	**0.5574**
	Random Forest	0.6167	0.5832	0.5432	0.5748	0.5198
	LGBMClassifier	0.6168	0.5717	0.5354	0.5607	0.5149
	AdaBoost	0.5883	0.5613	0.5333	0.5412	0.5312
dHOFC	Logistic regression	0.7278	0.661	0.6378	0.6567	0.6225
	KNN	0.6424	0.6105	0.523	0.6309	0.4537
	RidgeClassifier	0.5955	0.5588	0.5268	0.5474	0.512
	Naive_bayes_GaussianNB	0.7151	0.6505	0.6264	0.6455	0.6114
	Linear SVM	**0.7315**	**0.675**	**0.6641**	**0.6601**	**0.6706**
	Non-linear SVM	0.7216	0.6583	0.6415	0.6478	0.6383
	Random Forest	0.64	0.5925	0.5255	0.5973	0.4723
	LGBMClassifier	0.6762	0.6093	0.5601	0.6137	0.5202
	AdaBoost	0.6866	0.622	0.6004	0.6111	0.5953
SSGSR	Logistic regression	0.5619	0.5612	0.4546	0.5741	0.3834
	KNN	0.5954	0.5833	0.5183	0.5868	0.4693
	RidgeClassifier	0.6129	0.5638	0.2712	**0.7157**	0.1715
	Naive_bayes_GaussianNB	0.5556	0.5678	0.4425	0.5999	0.3566
	Linear SVM	0.5749	0.556	0.2562	0.6708	0.1633
	Non-linear SVM	0.6057	0.5845	0.436	0.6401	0.3353
	Random Forest	**0.6248**	**0.6103**	**0.5414**	0.6252	0.4827
	LGBMClassifier	0.5973	0.5728	0.531	0.5667	**0.5013**
	AdaBoost	0.5624	0.5676	0.5158	0.5603	0.4828
SLR	Logistic regression	0.559	0.542	0.5035	0.5291	0.4826
	KNN	0.5367	0.5239	0.3486	0.5124	0.2764
	RidgeClassifier	0.6085	0.569	0.4857	0.5763	0.4236
	Naive_bayes_GaussianNB	0.5452	0.5381	0.4819	0.528	0.4451
	Linear SVM	0.553	0.5445	0.505	0.53	0.4851
	Non-linear SVM	0.5973	0.5742	0.5511	0.5601	0.5471
	Random Forest	0.601	0.5767	0.5578	0.5653	0.5548
	LGBMClassifier	**0.6616**	**0.6219**	**0.5936**	**0.6182**	**0.5764**
	AdaBoost	0.5884	0.569	0.5517	0.559	0.5521
Fusion	Logistic regression	0.6736	0.6194	0.6019	0.6067	0.6003
	KNN	0.6305	0.5821	0.4157	0.6245	0.3221
	RidgeClassifier	0.6466	0.5703	0.3027	**0.6925**	0.2013
	Naive_bayes_GaussianNB	0.6709	0.6142	0.5753	0.6138	0.5442
	Linear SVM	0.6617	0.6091	0.5735	0.6062	0.5467
	Non-linear SVM	0.7228	0.6581	0.6409	0.65	0.6352
	Random Forest	0.6609	0.6064	0.5599	0.6012	0.5281
	LGBMClassifier	0.6936	0.6555	0.6193	0.6603	0.5871
	AdaBoost	0.6815	0.6244	0.5887	0.6174	0.5655
	MKL	**0.7408**	**0.6916**	**0.6743**	0.6887	**0.6622**

The bold values are the best classification metrics of all feature types.

### Biomarker identification

**Fig. 5:**
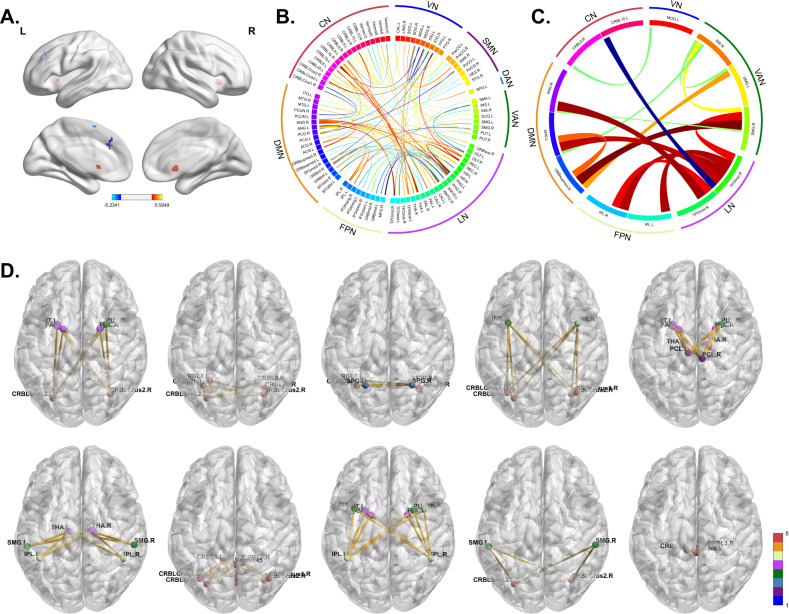
The identified biomarkers with significantly discriminative power. **A** The cluster map shows the results of the two-sample t-test with GRF correction on ALFF. The clusters with the peak activation strength (t-value) signed ‘+’ are colored in orange, and those signed ‘-’ are colored in blue; **B**, **C** The circos maps shows the abnormal brain region connectivity of SLR (**B**) and SSGSR (**C**). The ROIs of the AAL atlas are denoted on the inner circle, the corresponding brain networks [16] on the outer one. The width and color of links encode the t-value. The wider links correspond to the larger absolute t-values; the warm colors (red and orange) and cool colors (blue and cyan) correspond to the positive and negative t-value, respectively. **D** The selected top 10 dHOFC clusters of functional connectivity were drawn within the brain surface. The node color represents the functional network to which it belongs. VN visual network; SMN sensory-motor network; DAN dorsal attention network; VAN ventral attention network; LN limbic network; FPN frontoparietal network; DMN default mode network; CN cerebellum network.

We selected the voxels passing the two-sample t-test with GRF correction for voxel-wise ALFF as abnormal voxels, which formed 4 clusters shown in Fig. 5A and Supplemental Table 4. For the ROI-wise measures, we selected biomarkers (dHOFC: pairs of clusters, SSGSR, and SLR: FCs) that were always chosen in each CV fold by Boruta and passed t-test and FDR correction. Then, for dHOFC, we calculated the counts of each cluster that appeared in all pairs and chose the top 10 clusters of FCs as the final biomarkers (shown in Fig. 5D and Supplemental Table 5). For SLR and SSGSR, we sorted FCs according to their p-values and then selected the top-ranked 20 connections of SLR and the whole 15 discriminative connections of SSGSR (shown in Fig. 5B, C and Supplemental Table 6).

## Discussion

This study investigated the performances of different machine learning algorithms in classifying ADHD patients vs. healthy controls. To our knowledge, this is the first study that applied PC-derived tHOFC and dHOFC and SR-derived GSR, SSGSR, and SLR to diagnose ADHD based on the ABCD dataset. We also extracted the traditional fMRI-based measures (voxel-wise measures, PC, and SR) and compared their discriminative power. Our results showed that dHOFC achieved the best classification AUC of 0.7315, whose superior ability to identify ADHD patients suggests that the dynamic FC might underlie spontaneous fluctuations in attention [55, 59]. Notably, Wang et al. [55] achieved a higher AUC of 0.84 based on the measure of the temporal variability of dynamic functional connectivity, outperforming other traditional measures. However, the total sample size of 240 in their study is much smaller than ours (775). For ADHD, the reported classification model’s performance deteriorates with sample size [3, 4]. SLR resulted in sparsity but it preserved modularity structure in FC networks [45] and also achieved a high classification AUC of 0.6616 compared with other measures except for dHOFC. These two biologically meaningful measures provide complementary neuroimaging information for traditional indices.

The existing research on ADHD with rsfMRI is mainly based on the ADHD-200 dataset [46], collected from the subjects aged 7–27. However, only two published ADHD neuroimaging studies are based on the ABCD dataset. Owens et al. [60] considered the measures from sMRI and three task fMRI as predictors respectively, and used Elastic Net Regression to predict a continuous ADHD symptomatology coefficient. The best model was achieved on EN-Back features and explained 2.0% of the variance (R2 = 2.0%) in ADHD symptomatology regardless of covariates, while after all covariates are taken into account, R2 drops to 0.6%. Regarding the categorical analyses, they did not robustly predict the ADHD diagnosis from KSADS. Another study [40] comprehensively considered features from three modalities (sMRI, DTI, and resting-state fMRI) and finally reached an AUC of 0.698 using the Multiple Kernel Learning framework. Our study achieved an AUC of 0.7408, considering only resting-state fMRI, superior to the studies above. In addition, our results are better than most studies with similar sample sizes, only inferior to these two [61, 62] (detailed in Table 5).

**Table 5:** Published resting-state fMRI-based ADHD classification studies.

ACC	AUC	Number of subjects	Classifier	Dataset	References
0.6916	0.741	775	MKL	ABCD	Ours
0.688	0.70	776	Sparse LR	ADHD-200	Zhan et al. [73]
0.7884	-	645	T-R-SVM	ADHD-200	Shao et al. [62]
0.6491	-	729	SVM	ADHD-200	Sen et al. [74]
0.6604	-	730	CNN	ADHD-200	Zou et al. [36]
0.597	-	940	SVM	ADHD-200	Ghiassian et al. [75]
0.54	-	929	LR	ADHD-200	Sato et al. [30]
0.6667	-	839	SVM	ADHD-200	Sidhu et al. [76]
0.6959	-	947	PCA-LDA	ADHD-200	Dey et al. [61]

Our study found the aberrant neuroimaging biomarkers with significant discriminative power in identifying ADHD patients based on fMRI-derived measures. For voxel-based ALFF, the enhanced brain activities lay in the cerebellum and caudate nucleus of ADHD patients, whereas the decreased ones were found in the medial superior frontal gyrus and pre-motor and supplementary motor cortex. These regional brain aberrances are in line with previous findings [32, 33, 35, 63, 64]. The caudate nucleus and supplementary motor cortex are part of the executive control network, and these regions are involved in attention controls [65, 66], which also verifies our findings. For ROI-based dHOFC, SSGSR, and SLR, the number of increased FCs in ADHD was much more than that of the decreased ones. These abnormal FCs of brain regions were within and between cerebellum network (CN), limbic network (LN), ventral attention network (VAN), frontoparietal network (FPN), and default mode network (DMN). The connections from DMN to LN, CN, FPN, and VAN are much stronger in ADHD patients than in healthy controls, and similar tendencies were reported in the previous studies [67, 68]. Moreover, the connections from LN (left thalamus, left parahippocampal gyrus, left/right gyrus rectus, left pallidum, and right middle/superior temporal gyrus) to DMN, CN, and FPN are abnormal in ADHD. Earlier, the thalamus was reported as a mediator in frontostriatal circuitry during attention tasks [69]. These findings imply that DMN and LN might act as two core nodes interfering connections with the regions from other brain networks in ADHD. In addition, there are increased FCs within CN (left inferior cerebellum and right inferior cerebellum), LN (right gyrus rectus and left parahippocampal gyrus), DMN (left angular gyrus and right superior frontal gyrus), and VAN (right insula and left/right supramarginal gyrus), as well as decreased FCs within VN (right middle occipital gyrus and left inferior occipital gyrus) in ADHD. The insula can also be considered as part of the salience network, which plays a crucial role in attention [70]. In summary, the identified neuroimaging biomarkers in our study are widespread in brain regions, overlapping the findings in the previous literature discussed above. The measures provide complementary insights into understanding the underlying ADHD pathogenesis and its diagnosis from multiple perspectives.

This study has two limitations. One is that we only adopted the classic machine learning algorithms. Deep learning models (e.g., deep models for image preprocessing and classification [71, 72]) that can automatically extract high-level and compact feature representations will be introduced in the future. Another is that we performed binary classification of patients/controls but neglected the ADHD subtypes.

## Conclusion

This study proposed an automated ADHD classification framework on a multi-site ABCD dataset containing children aged between 9 and 10. We extracted voxel-wise and ROI-wise quantitative measures from resting-state fMRI and used several machine learning methods to predict the ADHD diagnosis. Classification models on ROI-wise dHOFC and SLR outperformed other measures owing to their more biologically meaningful and complementary neuroimaging information. The highest classification AUC was achieved using MKL by fusing different features. The identified aberrant regions (cerebellum, caudate nucleus, medial superior frontal gyrus, pre-motor and supplementary motor cortex) and FCs (within and between CN, LN, VAN, FPN, and DMN) are widespread in the whole brain and conform with previous literature generally.

## Supplementary information

Supplemental material

## Data Availability

We used publicly available MATLAB-based tools (DPARSF v5.1 and BrainNetClass toolbox v1.1) to implement the calculation of measures. All codes used to extract measures and generate results that are reported in this paper are available upon request.
