# Supplementary material for: Multiple measurement analysis of resting-state fMRI for ADHD classification in adolescent brain from the ABCD study
Source: Transl Psychiatry. 2023 Feb 6;13:45. doi: 10.1038/s41398-023-02309-5 (PMC9902465; doi:10.1038/s41398-023-02309-5)
Supplement: Supplementary file 1 — Supplemental material [file 41398_2023_2309_MOESM1_ESM.docx]

**Supplementary Material**

**Multi-modal classification.**

We applied two multi-modal classification methods (model-agnostic early fusion strategy and model-based Multiple Kernel Learning (MKL) algorithm^1, 2^) to fusing the discriminative features from different modalities (ALFF, dHOFC, SSGSR, and SLR) and improving the classification performance.

**Model-agnostic early fusion strategy**

We simply concatenated the multi-modal measures, and then applied nine classification algorithms (detailed in the Materials and Methods 2.4 section) to testing the performance.

**Model-based MKL algorithm**

The SVM-based MKL was applied in this study, and the dual form of MKL-SVM was defined as:

$$\min_{\alpha} \frac{1}{2}\sum_{i=1}^{N} \sum_{j=1}^{N} \alpha_{i}\alpha_{j}y_{i}y_{j}\left[ \sum_{m=1}^{M} w_{m}K\left( x_{i}^{m},x_{j}^{m} \right) \right]-\sum_{i=1}^{N} \alpha_{i}$$

$$\begin{aligned} s.t. \sum_{i=1}^{N} \alpha_{i}y_{i}=0, 0\leq\alpha_{i}\leq C, i=1,\ldots,N\#\left( AUTONUM \backslash* Arabic \right) \end{aligned}$$

where $x_{i}^{m}$ is the $i^{th}$ subject’s measure of the $m^{th}$ modality, M is the number of modalities, $K\left( x_{i}^{m},x_{j}^{m} \right)$ is the kernel function, $y_{i}$ is the label of the $i^{th}$ subject, $\alpha_{i}$ is the Lagrange multiplier introduced by dual algorithm, $w_{m}$ is the weight assigned to the kernel corresponding to the $m^{th}$ modality, in our case, M=4 and N=775. We solved the problem by iteratively optimizing $\alpha_{i}$ and $w_{m}$. The decision function was formulated as:

$$\begin{aligned} f\left( x \right)=sign\left( \sum_{i=1}^{N} \alpha_{i}y_{i}\sum_{m=1}^{M} w_{m}K\left( x_{i}^{m},x_{j}^{m} \right)+b \right)\#\left( AUTONUM \backslash* Arabic \right) \end{aligned}$$

where $b=y_{j}-\sum_{i=1}^{N} \alpha_{i}y_{i}\sum_{m=1}^{M} w_{m}K\left( x_{i}^{m},x_{j}^{m} \right)$. In this study, we used the Python-based EasyMKL^3^ to implement the MKL-SVM classification.

**Supplemental Table 1.** **Remaining subjects after each exclusion step**

|  | **Case** | **Control** |
| --- | --- | --- |
| **Original labels** | 1444 | 1859 |
| **Recommended MRI inclusion criteria** | 612 | 1337 |
| **No** **hydrocephalus nor herniation** | 612 | 1337 |
| **Right-handedness** | 575 | 1246 |
| **Improbable or possible mild TBI** | 564 | 1238 |
| **No missing covariates** | 548 | 1187 |
| **Phillips scanner removal** | 493 | 1067 |
| **Downsampling and QC for normalization** | 410 | 438 |
| **QC for head motion** | 373 | 402 |

**Supplemental Table 2. Brain ROIs of the AAL atlas**

| ID | AAL ROI name | Abbreviation | Coordinates | | |
| --- | --- | --- | --- | --- | --- |
|  |  |  | x | y | z |
| 1 | Precentral_L | PreCG.L | -38.65 | -5.68 | 50.94 |
| 2 | Precentral_R | PreCG.R | 41.37 | -8.21 | 52.09 |
| 3 | Frontal_Sup_L | SFGdor.L | -18.45 | 34.81 | 42.2 |
| 4 | Frontal_Sup_R | SFGdor.R | 21.9 | 31.12 | 43.82 |
| 5 | Frontal_Sup_Orb_L | ORBsup.L | -16.56 | 47.32 | -13.31 |
| 6 | Frontal_Sup_Orb_R | ORBsup.R | 18.49 | 48.1 | -14.02 |
| 7 | Frontal_Mid_L | MFG.L | -33.43 | 32.73 | 35.46 |
| 8 | Frontal_Mid_R | MFG.R | 37.59 | 33.06 | 34.04 |
| 9 | Frontal_Mid_Orb_L | ORBmid.L | -30.65 | 50.43 | -9.62 |
| 10 | Frontal_Mid_Orb_R | ORBmid.R | 33.18 | 52.59 | -10.73 |
| 11 | Frontal_Inf_Oper_L | IFGoperc.L | -48.43 | 12.73 | 19.02 |
| 12 | Frontal_Inf_Oper_R | IFGoperc.R | 50.2 | 14.98 | 21.41 |
| 13 | Frontal_Inf_Tri_L | IFGtriang.L | -45.58 | 29.91 | 13.99 |
| 14 | Frontal_Inf_Tri_R | IFGtriang.R | 50.33 | 30.16 | 14.17 |
| 15 | Frontal_Inf_Orb_L | ORBinf.L | -35.98 | 30.71 | -12.11 |
| 16 | Frontal_Inf_Orb_R | ORBinf.R | 41.22 | 32.23 | -11.91 |
| 17 | Rolandic_Oper_L | ROL.L | -47.16 | -8.48 | 13.95 |
| 18 | Rolandic_Oper_R | ROL.R | 52.65 | -6.25 | 14.63 |
| 19 | Supp_Motor_Area_L | SMA.L | -5.32 | 4.85 | 61.38 |
| 20 | Supp_Motor_Area_R | SMA.R | 8.62 | 0.17 | 61.85 |
| 21 | Olfactory_L | OLF.L | -8.06 | 15.05 | -11.46 |
| 22 | Olfactory_R | OLF.R | 10.43 | 15.91 | -11.26 |
| 23 | Frontal_Sup_Medial_L | SFGmed.L | -4.8 | 49.17 | 30.89 |
| 24 | Frontal_Sup_Medial_R | SFGmed.R | 9.1 | 50.84 | 30.22 |
| 25 | Frontal_Med_Orb_L | ORBsupmed.L | -5.17 | 54.06 | -7.4 |
| 26 | Frontal_Med_Orb_R | ORBsupmed.R | 8.16 | 51.67 | -7.13 |
| 27 | Rectus_L | REC.L | -5.08 | 37.07 | -18.14 |
| 28 | Rectus_R | REC.R | 8.35 | 35.64 | -18.04 |
| 29 | Insula_L | INS.L | -35.13 | 6.65 | 3.44 |
| 30 | Insula_R | INS.R | 39.02 | 6.25 | 2.08 |
| 31 | Cingulum_Ant_L | ACG.L | -4.04 | 35.4 | 13.95 |
| 32 | Cingulum_Ant_R | ACG.R | 8.46 | 37.01 | 15.84 |
| 33 | Cingulum_Mid_L | DCG.L | -5.48 | -14.92 | 41.57 |
| 34 | Cingulum_Mid_R | DCG.R | 8.02 | -8.83 | 39.79 |
| 35 | Cingulum_Post_L | PCG.L | -4.85 | -42.92 | 24.67 |
| 36 | Cingulum_Post_R | PCG.R | 7.44 | -41.81 | 21.87 |
| 37 | Hippocampus_L | HIP.L | -25.03 | -20.74 | -10.13 |
| 38 | Hippocampus_R | HIP.R | 29.23 | -19.78 | -10.33 |
| 39 | ParaHippocampal_L | PHG.L | -21.17 | -15.95 | -20.7 |
| 40 | ParaHippocampal_R | PHG.R | 25.38 | -15.15 | -20.47 |
| 41 | Amygdala_L | AMYG.L | -23.27 | -0.67 | -17.14 |
| 42 | Amygdala_R | AMYG.R | 27.32 | 0.64 | -17.5 |
| 43 | Calcarine_L | CAL.L | -7.14 | -78.67 | 6.44 |
| 44 | Calcarine_R | CAL.R | 15.99 | -73.15 | 9.4 |
| 45 | Cuneus_L | CUN.L | -5.93 | -80.13 | 27.22 |
| 46 | Cuneus_R | CUN.R | 13.51 | -79.36 | 28.23 |
| 47 | Lingual_L | LING.L | -14.62 | -67.56 | -4.63 |
| 48 | Lingual_R | LING.R | 16.29 | -66.93 | -3.87 |
| 49 | Occipital_Sup_L | SOG.L | -16.54 | -84.26 | 28.17 |
| 50 | Occipital_Sup_R | SOG.R | 24.29 | -80.85 | 30.59 |
| 51 | Occipital_Mid_L | MOG.L | -32.39 | -80.73 | 16.11 |
| 52 | Occipital_Mid_R | MOG.R | 37.39 | -79.7 | 19.42 |
| 53 | Occipital_Inf_L | IOG.L | -36.36 | -78.29 | -7.84 |
| 54 | Occipital_Inf_R | IOG.R | 38.16 | -81.99 | -7.61 |
| 55 | Fusiform_L | FFG.L | -31.16 | -40.3 | -20.23 |
| 56 | Fusiform_R | FFG.R | 33.97 | -39.1 | -20.18 |
| 57 | Postcentral_L | PoCG.L | -42.46 | -22.63 | 48.92 |
| 58 | Postcentral_R | PoCG.R | 41.43 | -25.49 | 52.55 |
| 59 | Parietal_Sup_L | SPG.L | -23.45 | -59.56 | 58.96 |
| 60 | Parietal_Sup_R | SPG.R | 26.11 | -59.18 | 62.06 |
| 61 | Parietal_Inf_L | IPL.L | -42.8 | -45.82 | 46.74 |
| 62 | Parietal_Inf_R | IPL.R | 46.46 | -46.29 | 49.54 |
| 63 | SupraMarginal_L | SMG.L | -55.79 | -33.64 | 30.45 |
| 64 | SupraMarginal_R | SMG.R | 57.61 | -31.5 | 34.48 |
| 65 | Angular_L | ANG.L | -44.14 | -60.82 | 35.59 |
| 66 | Angular_R | ANG.R | 45.51 | -59.98 | 38.63 |
| 67 | Precuneus_L | PCUN.L | -7.24 | -56.07 | 48.01 |
| 68 | Precuneus_R | PCUN.R | 9.98 | -56.05 | 43.77 |
| 69 | Paracentral_Lobule_L | PCL.L | -7.63 | -25.36 | 70.07 |
| 70 | Paracentral_Lobule_R | PCL.R | 7.48 | -31.59 | 68.09 |
| 71 | Caudate_L | CAU.L | -11.46 | 11 | 9.24 |
| 72 | Caudate_R | CAU.R | 14.84 | 12.07 | 9.42 |
| 73 | Putamen_L | PUT.L | -23.91 | 3.86 | 2.4 |
| 74 | Putamen_R | PUT.R | 27.78 | 4.91 | 2.46 |
| 75 | Pallidum_L | PAL.L | -17.75 | -0.03 | 0.21 |
| 76 | Pallidum_R | PAL.R | 21.2 | 0.18 | 0.23 |
| 77 | Thalamus_L | THA.L | -10.85 | -17.56 | 7.98 |
| 78 | Thalamus_R | THA.R | 13 | -17.55 | 8.09 |
| 79 | Heschl_L | HES.L | -41.99 | -18.88 | 9.98 |
| 80 | Heschl_R | HES.R | 45.86 | -17.15 | 10.41 |
| 81 | Temporal_Sup_L | STG.L | -53.16 | -20.68 | 7.13 |
| 82 | Temporal_Sup_R | STG.R | 58.15 | -21.78 | 6.8 |
| 83 | Temporal_Pole_Sup_L | TPOsup.L | -39.88 | 15.14 | -20.18 |
| 84 | Temporal_Pole_Sup_R | TPOsup.R | 48.25 | 14.75 | -16.86 |
| 85 | Temporal_Mid_L | MTG.L | -55.52 | -33.8 | -2.2 |
| 86 | Temporal_Mid_R | MTG.R | 57.47 | -37.23 | -1.47 |
| 87 | Temporal_Pole_Mid_L | TPOmid.L | -36.32 | 14.59 | -34.08 |
| 88 | Temporal_Pole_Mid_R | TPOmid.R | 44.22 | 14.55 | -32.23 |
| 89 | Temporal_Inf_L | ITG.L | -49.77 | -28.05 | -23.17 |
| 90 | Temporal_Inf_R | ITG.R | 53.69 | -31.07 | -22.32 |
| 91 | Cerebelum_Crus1_L | CRBLCrus1.L | -36.07 | -66.72 | -28.93 |
| 92 | Cerebelum_Crus1_R | CRBLCrus1.R | 37.46 | -67.14 | -29.55 |
| 93 | Cerebelum_Crus2_L | CRBLCrus2.L | -28.64 | -73.26 | -38.2 |
| 94 | Cerebelum_Crus2_R | CRBLCrus2.R | 32.06 | -69.02 | -39.95 |
| 95 | Cerebelum_3_L | CRBL3.L | -8.8 | -37.22 | -18.58 |
| 96 | Cerebelum_3_R | CRBL3.R | 12.32 | -34.47 | -19.39 |
| 97 | Cerebelum_4_5_L | CRBL45.L | -15 | -43.49 | -16.93 |
| 98 | Cerebelum_4_5_R | CRBL45.R | 17.2 | -42.86 | -18.15 |
| 99 | Cerebelum_6_L | CRBL6.L | -23.24 | -59.1 | -22.13 |
| 100 | Cerebelum_6_R | CRBL6.R | 24.69 | -58.32 | -23.65 |
| 101 | Cerebelum_7b_L | CRBL7b.L | -32.36 | -59.82 | -45.45 |
| 102 | Cerebelum_7b_R | CRBL7b.R | 33.14 | -63.18 | -48.46 |
| 103 | Cerebelum_8_L | CRBL8.L | -25.75 | -54.52 | -47.68 |
| 104 | Cerebelum_8_R | CRBL8.R | 25.06 | -56.34 | -49.47 |
| 105 | Cerebelum_9_L | CRBL9.L | -10.95 | -48.95 | -45.9 |
| 106 | Cerebelum_9_R | CRBL9.R | 9.46 | -49.5 | -46.33 |
| 107 | Cerebelum_10_L | CRBL10.L | -22.61 | -33.8 | -41.76 |
| 108 | Cerebelum_10_R | CRBL10.R | 25.99 | -33.84 | -41.35 |
| 109 | Vermis_1_2 | Vermis12 | 0.76 | -38.79 | -20.05 |
| 110 | Vermis_3 | Vermis3 | 1.38 | -39.93 | -11.4 |
| 111 | Vermis_4_5 | Vermis45 | 1.22 | -52.36 | -6.11 |
| 112 | Vermis_6 | Vermis6 | 1.14 | -67.06 | -15.12 |
| 113 | Vermis_7 | Vermis7 | 1.15 | -71.93 | -25.14 |
| 114 | Vermis_8 | Vermis8 | 1.15 | -64.43 | -34.08 |
| 115 | Vermis_9 | Vermis9 | 0.86 | -54.87 | -34.9 |
| 116 | Vermis_10 | Vermis10 | 0.36 | -45.8 | -31.68 |

**Supplemental Table 3. Classification performance**

| **Feature type** | **Classifier** | **AUC** | **ACC** | **F1** | **Precision** | **Recall** |
| --- | --- | --- | --- | --- | --- | --- |
| **ALFF** | **Logistic Regression** | 0.5668 | 0.5446 | 0.5157 | 0.529 | 0.5063 |
|  | **KNN** | 0.6007 | 0.5613 | 0.3543 | 0.6043 | 0.2574 |
|  | **RidgeClassifier** | 0.5724 | 0.5536 | 0.5159 | 0.5388 | 0.4983 |
|  | **Naive_bayes_GaussianNB** | 0.6183 | **0.5921** | 0.4746 | **0.627** | 0.3888 |
|  | **Linear SVM** | 0.5588 | 0.5446 | 0.515 | 0.5272 | 0.5066 |
|  | **Non-linear SVM** | **0.624** | 0.5819 | **0.5599** | 0.5662 | **0.5574** |
|  | **Random Forest** | 0.6167 | 0.5832 | 0.5432 | 0.5748 | 0.5198 |
|  | **LGBMClassifier** | 0.6168 | 0.5717 | 0.5354 | 0.5607 | 0.5149 |
|  | **AdaBoost** | 0.5883 | 0.5613 | 0.5333 | 0.5412 | 0.5312 |
| **ReHo** | **Logistic Regression** | 0.551 | 0.5418 | 0.5155 | 0.5218 | 0.5118 |
|  | **KNN** | 0.5958 | 0.5679 | 0.4521 | 0.5855 | 0.3778 |
|  | **RidgeClassifier** | 0.5571 | 0.5405 | 0.5005 | 0.5243 | 0.4821 |
|  | **Naive_bayes_GaussianNB** | 0.5862 | 0.5536 | 0.3779 | 0.5527 | 0.3034 |
|  | **Linear SVM** | 0.5409 | 0.5315 | 0.5002 | 0.5141 | 0.4903 |
|  | **Non-linear SVM** | 0.5685 | 0.5419 | 0.5192 | 0.5235 | 0.5171 |
|  | **Random Forest** | 0.5884 | 0.5871 | 0.5353 | 0.5839 | 0.4982 |
|  | **LGBMClassifier** | 0.5869 | 0.5664 | 0.5396 | 0.5483 | 0.5331 |
|  | **AdaBoost** | 0.5632 | 0.5353 | 0.5179 | 0.5192 | 0.52 |
| **fALFF** | **Logistic Regression** | 0.5615 | 0.5586 | 0.5338 | 0.5441 | 0.5257 |
|  | **KNN** | 0.6021 | 0.5601 | 0.3797 | 0.586 | 0.29 |
|  | **RidgeClassifier** | 0.5669 | 0.5626 | 0.5215 | 0.5525 | 0.4962 |
|  | **Naive_bayes_GaussianNB** | 0.5903 | 0.5625 | 0.4641 | 0.5708 | 0.3991 |
|  | **Linear SVM** | 0.5709 | 0.569 | 0.5249 | 0.5587 | 0.4961 |
|  | **Non-linear SVM** | 0.606 | 0.5846 | 0.5513 | 0.5745 | 0.5338 |
|  | **Random Forest** | 0.6043 | 0.5754 | 0.5347 | 0.5652 | 0.5092 |
|  | **LGBMClassifier** | 0.5863 | 0.5626 | 0.5161 | 0.5512 | 0.4884 |
|  | **AdaBoost** | 0.5613 | 0.5432 | 0.5001 | 0.5243 | 0.4828 |
| **PC** | **Logistic Regression** | 0.5499 | 0.5459 | 0.5031 | 0.5333 | 0.4802 |
|  | **KNN** | 0.5126 | 0.5084 | 0.3717 | 0.4765 | 0.3085 |
|  | **RidgeClassifier** | 0.5337 | 0.5304 | 0.5155 | 0.513 | 0.5204 |
|  | **Naive_bayes_GaussianNB** | 0.547 | 0.5306 | 0.5046 | 0.5127 | 0.4989 |
|  | **Linear SVM** | 0.5449 | 0.537 | 0.5081 | 0.5214 | 0.499 |
|  | **Non-linear SVM** | 0.5298 | 0.5124 | 0.4799 | 0.4911 | 0.4721 |
|  | **Random Forest** | 0.5453 | 0.5433 | 0.4831 | 0.5346 | 0.4454 |
|  | **LGBMClassifier** | 0.5369 | 0.524 | 0.4893 | 0.508 | 0.4749 |
|  | **AdaBoost** | 0.5351 | 0.5356 | 0.5092 | 0.5185 | 0.5064 |
| **tHOFC** | **Logistic Regression** | 0.5127 | 0.5058 | 0.4524 | 0.4857 | 0.429 |
|  | **KNN** | 0.4987 | 0.52 | 0.4235 | 0.4989 | 0.3751 |
|  | **RidgeClassifier** | 0.5219 | 0.5071 | 0.4752 | 0.4876 | 0.4664 |
|  | **Naive_bayes_GaussianNB** | 0.525 | 0.5201 | 0.4958 | 0.4993 | 0.496 |
|  | **Linear SVM** | 0.5011 | 0.5123 | 0.4747 | 0.4946 | 0.4609 |
|  | **Non-linear SVM** | 0.5254 | 0.5316 | 0.4835 | 0.5136 | 0.4613 |
|  | **Random Forest** | 0.5188 | 0.5173 | 0.4582 | 0.4979 | 0.4263 |
|  | **LGBMClassifier** | 0.4954 | 0.515 | 0.4885 | 0.495 | 0.4856 |
|  | **AdaBoost** | 0.4759 | 0.498 | 0.4589 | 0.4785 | 0.4447 |
| **dHOFC** | **Logistic Regression** | 0.7278 | 0.661 | 0.6378 | 0.6567 | 0.6225 |
|  | **KNN** | 0.6424 | 0.6105 | 0.523 | 0.6309 | 0.4537 |
|  | **RidgeClassifier** | 0.5955 | 0.5588 | 0.5268 | 0.5474 | 0.512 |
|  | **Naive_bayes_GaussianNB** | 0.7151 | 0.6505 | 0.6264 | 0.6455 | 0.6114 |
|  | **Linear SVM** | 0.7315 | 0.675 | 0.6641 | 0.6601 | 0.6706 |
|  | **Non-linear SVM** | 0.7216 | 0.6583 | 0.6415 | 0.6478 | 0.6383 |
|  | **Random Forest** | 0.64 | 0.5925 | 0.5255 | 0.5973 | 0.4723 |
|  | **LGBMClassifier** | 0.6762 | 0.6093 | 0.5601 | 0.6137 | 0.5202 |
|  | **AdaBoost** | 0.6866 | 0.622 | 0.6004 | 0.6111 | 0.5953 |
| **SR** | **Logistic Regression** | 0.546 | 0.5638 | 0.4624 | 0.5703 | 0.3945 |
|  | **KNN** | 0.534 | 0.5329 | 0.4739 | 0.5241 | 0.4371 |
|  | **RidgeClassifier** | 0.5249 | 0.5174 | 0.0412 | 0.2222 | 0.0242 |
|  | **Naive_bayes_GaussianNB** | 0.5085 | 0.5251 | 0.2316 | 0.4083 | 0.1825 |
|  | **Linear SVM** | 0.5386 | 0.5612 | 0.4237 | 0.5839 | 0.3434 |
|  | **Non-linear SVM** | 0.5535 | 0.5407 | 0.384 | 0.5513 | 0.3119 |
|  | **Random Forest** | 0.5706 | 0.5806 | 0.4904 | 0.5939 | 0.424 |
|  | **LGBMClassifier** | 0.5727 | 0.5586 | 0.5253 | 0.55 | 0.5098 |
|  | **AdaBoost** | 0.5607 | 0.5598 | 0.4797 | 0.5577 | 0.4237 |
| **GSR** | **Logistic Regression** | 0.5538 | 0.5651 | 0.3404 | 0.6467 | 0.2442 |
|  | **KNN** | 0.5836 | 0.5419 | 0.4754 | 0.5305 | 0.4402 |
|  | **RidgeClassifier** | 0.5955 | 0.5806 | 0.3945 | 0.6482 | 0.287 |
|  | **Naive_bayes_GaussianNB** | 0.5455 | 0.569 | 0.3075 | 0.5982 | 0.2146 |
|  | **Linear SVM** | 0.546 | 0.5407 | 0.1463 | 0.7069 | 0.086 |
|  | **Non-linear SVM** | 0.6218 | 0.578 | 0.4414 | 0.6125 | 0.3566 |
|  | **Random Forest** | 0.6107 | 0.5843 | 0.5544 | 0.5712 | 0.5467 |
|  | **LGBMClassifier** | 0.5828 | 0.5574 | 0.5349 | 0.547 | 0.5312 |
|  | **AdaBoost** | 0.5819 | 0.5457 | 0.4996 | 0.5333 | 0.4935 |
| **SSGSR** | **Logistic Regression** | 0.5619 | 0.5612 | 0.4546 | 0.5741 | 0.3834 |
|  | **KNN** | 0.5954 | 0.5833 | 0.5183 | 0.5868 | 0.4693 |
|  | **RidgeClassifier** | 0.6129 | 0.5638 | 0.2712 | 0.7157 | 0.1715 |
|  | **Naive_bayes_GaussianNB** | 0.5556 | 0.5678 | 0.4425 | 0.5999 | 0.3566 |
|  | **Linear SVM** | 0.5749 | 0.556 | 0.2562 | 0.6708 | 0.1633 |
|  | **Non-linear SVM** | 0.6057 | 0.5845 | 0.436 | 0.6401 | 0.3353 |
|  | **Random Forest** | 0.6248 | 0.6103 | 0.5414 | 0.6252 | 0.4827 |
|  | **LGBMClassifier** | 0.5973 | 0.5728 | 0.531 | 0.5667 | 0.5013 |
|  | **AdaBoost** | 0.5624 | 0.5676 | 0.5158 | 0.5603 | 0.4828 |
| **SLR** | **Logistic Regression** | 0.559 | 0.542 | 0.5035 | 0.5291 | 0.4826 |
|  | **KNN** | 0.5367 | 0.5239 | 0.3486 | 0.5124 | 0.2764 |
|  | **RidgeClassifier** | 0.6085 | 0.569 | 0.4857 | 0.5763 | 0.4236 |
|  | **Naive_bayes_GaussianNB** | 0.5452 | 0.5381 | 0.4819 | 0.528 | 0.4451 |
|  | **Linear SVM** | 0.553 | 0.5445 | 0.505 | 0.53 | 0.4851 |
|  | **Non-linear SVM** | 0.5973 | 0.5742 | 0.5511 | 0.5601 | 0.5471 |
|  | **Random Forest** | 0.601 | 0.5767 | 0.5578 | 0.5653 | 0.5548 |
|  | **LGBMClassifier** | 0.6616 | 0.6219 | 0.5936 | 0.6182 | 0.5764 |
|  | **AdaBoost** | 0.5884 | 0.569 | 0.5517 | 0.559 | 0.5521 |
| **Fusion** | **Logistic Regression** | 0.6736 | 0.6194 | 0.6019 | 0.6067 | 0.6003 |
|  | **KNN** | 0.6305 | 0.5821 | 0.4157 | 0.6245 | 0.3221 |
|  | **RidgeClassifier** | 0.6466 | 0.5703 | 0.3027 | 0.6925 | 0.2013 |
|  | **Naive_bayes_GaussianNB** | 0.6709 | 0.6142 | 0.5753 | 0.6138 | 0.5442 |
|  | **Linear SVM** | 0.6617 | 0.6091 | 0.5735 | 0.6062 | 0.5467 |
|  | **Non-linear SVM** | 0.7228 | 0.6581 | 0.6409 | 0.65 | 0.6352 |
|  | **Random Forest** | 0.6609 | 0.6064 | 0.5599 | 0.6012 | 0.5281 |
|  | **LGBMClassifier** | 0.6936 | 0.6555 | 0.6193 | 0.6603 | 0.5871 |
|  | **AdaBoost** | 0.6815 | 0.6244 | 0.5887 | 0.6174 | 0.5655 |
|  | **MKL** | 0.7408 | 0.6916 | 0.6743 | 0.6887 | 0.6622 |

**Supplemental Table 4. ALFF clusters with significant differences between two groups**

| **Cluster** | **Size** | **Peak coordinates** | | | **Peak activation strength (t)** | **Peak brain region** | **Other regions** |
| --- | --- | --- | --- | --- | --- | --- | --- |
|  |  | x | y | z |  |  |  |
| Cluster 1 | 700 | -3 | -81 | -30 | 5.50488 | CRBLCrus2.L | CRBLCrus1.L, CRBLCrus1.R, CRBL6.L, CRBL6.R |
| Cluster 2 | 190 | 18 | 24 | -3 | 5.20857 | CAU.R | CAU.L, PUT.R, OLF.R |
| Cluster 3 | 99 | 0 | 30 | 63 | -5.2341 | SFGmed.L | SMA.L, SMA.R, SFGmed.R |
| Cluster 4 | 84 | -9 | 6 | 60 | -5.0457 | BA6_L (Brodmann) | SFGdor.L, SFGmed.L |

**Supplemental Table 5. Top 10 dHOFC clusters with significantly discriminative power**

| **Cluster** | **Brain region connection** | **Network** |
| --- | --- | --- |
| Cluster 1 | (PUT.L, CRBLCrus2.L), (PUT.L, CRBLCrus2.R), (PUT.R, CRBLCrus2.L), (PUT.R, CRBLCrus2.R), (PAL.L, CRBLCrus2.L), (PAL.L, CRBLCrus2.R), (PAL.R, CRBLCrus2.L), (PAL.R, CRBLCrus2.R) | VAN, LN, CN |
| Cluster 2 | (CRBLCrus2.L, CRBL7b.L), (CRBLCrus2.L, CRBL7b.R), (CRBLCrus2.L, CRBL8.L), (CRBLCrus2.L, CRBL8.R), (CRBLCrus2.R, CRBL7b.L), (CRBLCrus2.R, CRBL7b.R), (CRBLCrus2.R, CRBL8.L), (CRBLCrus2.R, CRBL8.R) | CN |
| Cluster 3 | (SPG.L, CRBL7b.L), (SPG.L, CRBL7b.R), (SPG.L, CRBL8.L), (SPG.L, CRBL8.R), (SPG.R, CRBL7b.L), (SPG.R, CRBL7b.R), (SPG.R, CRBL8.L), (SPG.R, CRBL8.R) | DAN, CN |
| Cluster 4 | (INS.L, CRBLCrus1.L), (INS.L, CRBLCrus1.R), (INS.L, CRBLCrus2.L), (INS.L, CRBLCrus2.R), (INS.R, CRBLCrus1.L), (INS.R, CRBLCrus1.R), (INS.R, CRBLCrus2.L), (INS.R, CRBLCrus2.R) | VAN, CN |
| Cluster 5 | (PCL.L, PUT.L), (PCL.L, PUT.R), (PCL.L, PAL.L), (PCL.L, PAL.R), (PCL.L, THA.L), (PCL.L, THA.R), (PCL.R, PUT.L), (PCL.R, PUT.R), (PCL.R, PAL.L), (PCL.R, PAL.R), (PCL.R, THA.L), (PCL.R, THA.R) | SMN, VAN, FPN |
| Cluster 6 | (IPL.L, THA.L), (IPL.L, THA.R), (IPL.R, THA.L), (IPL.R, THA.R), (SMG.L, THA.L), (SMG.L, THA.R), (SMG.R, THA.L), (SMG.R, THA.R) | VAN, LN, FPN |
| Cluster 7 | (CRBLCrus1.L, CRBL9.L), (CRBLCrus1.L, CRBL9.R), (CRBLCrus1.R, CRBL9.L), (CRBLCrus1.R, CRBL9.R), (CRBLCrus2.L, CRBL9.L), (CRBLCrus2.L, CRBL9.R), (CRBLCrus2.R, CRBL9.L), (CRBLCrus2.R, CRBL9.R), (CRBL9.L, Vermis45), (CRBL9.L, Vermis6), (CRBL9.L, Vermis7), (CRBL9.R, Vermis45), (CRBL9.R, Vermis6), (CRBL9.R, Vermis7) | CN |
| Cluster 8 | (INS.L, IPL.L), (INS.L, IPL.R), (INS.R, IPL.L), (INS.R, IPL.R), (IPL.L, PUT.L), (IPL.L, PUT.R), (IPL.L, PAL.L), (IPL.L, PAL.R), (IPL.R, PUT.L), (IPL.R, PUT.R), (IPL.R, PAL.L), (IPL.R, PAL.R) | VAN, LN, FPN |
| Cluster 9 | (SMG.L, CRBLCrus2.L), (SMG.L, CRBLCrus2.R), (SMG.R, CRBLCrus2.L), (SMG.R, CRBLCrus2.R) | VAN, CN |
| Cluster 10 | (CRBL3.L, CRBL3.R), (CRBL3.L, Vermis12), (CRBL3.L, Vermis3), (CRBL3.R, Vermis12), (CRBL3.R, Vermis3) | CN |

**Supplemental Table 6. Brain region connections with significantly discriminative power**

| **Measure** | **Brain region connection** | **t-value** | **p-value** | **Brain region connection** | **t-value** | **p-value** |
| --- | --- | --- | --- | --- | --- | --- |
| SLR | (REC.R, PHG.L) | 4.6449 | 4.00E-06 | (ANG.L, TPOmid.R) | 4.6181 | 4.54E-06 |
|  | (THA.L, CRBL7b.R) | 4.4377 | 1.04E-05 | (ACG.L, ANG.R) | 4.2788 | 2.12E-05 |
|  | (PHG.L, ANG.R) | 4.1603 | 3.53E-05 | (REC.R, FFG.L) | 4.0762 | 5.05E-05 |
|  | (CRBLCrus2.R, CRBL10.L) | 4.0512 | 5.61E-05 | (PHG.L, CRBL7b.R) | 3.9173 | 9.75E-05 |
|  | (REC.L, PHG.L) | 3.8558 | 1.25E-04 | (THA.L, CRBL8.R) | 3.8221 | 1.43E-04 |
|  | (THA.L, CRBL8.L) | 3.7942 | 1.60E-04 | (PAL.L, CRBL7b.L) | 3.7898 | 1.62E-04 |
|  | (MFG.R, AMYG.L) | -3.7061 | 2.26E-04 | (REC.L, FFG.L) | 3.5838 | 3.60E-04 |
|  | (MOG.R, IOG.L) | -3.5572 | 3.98E-04 | (ACG.R, IPL.R) | 3.5421 | 4.21E-04 |
|  | (ORBinf.R, IPL.R) | 3.5261 | 4.47E-04 | (OLF.R, IPL.R) | 3.5153 | 4.65E-04 |
|  | (OLF.R, ANG.R) | 3.4967 | 4.98E-04 | (MOG.L, TPOsup.L) | 3.4916 | 5.07E-04 |
| SSGSR | (ORBsupmed.R, SMG.R) | 4.0547 | 5.53E-05 | (ANG.R, TPOmid.R) | 3.9924 | 7.16E-05 |
|  | (IPL.R, TPOsup.R) | 3.9323 | 9.17E-05 | (IPL.L, TPOsup.R) | 3.9285 | 9.31E-05 |
|  | (ANG.L, TPOsup.R) | 3.885 | 1.11E-04 | (IPL.R, TPOmid.R) | 3.7519 | 1.89E-04 |
|  | (SMG.R, TPOsup.R) | 3.7406 | 1.97E-04 | (ORBsupmed.R, ANG.L) | 3.45 | 5.91E-04 |
|  | (TPOsup.R, CRBL10.L) | -3.3187 | 9.47E-04 | (ORBsupmed.R, SMG.L) | 3.2333 | 1.28E-03 |
|  | (INS.R, SMG.R) | 2.9906 | 2.87E-03 | (INS.R, SMG.L) | 2.6716 | 7.71E-03 |
|  | (MOG.L, CRBL8.R) | 2.6158 | 9.08E-03 | (INS.R, ANG.L) | 2.5939 | 9.67E-03 |
|  | (SMG.R, ANG.R) | -2.3932 | 1.69E-02 |  |  |  |

**Supplemental References**

1. Gonen M, Alpaydin E. Multiple Kernel Learning Algorithms. *J Mach Learn Res* 2011; **12:** 2211-2268.

2. Lauriola I, Aiolli F. MKLpy: a python-based framework for Multiple Kernel Learning. *arXiv preprint arXiv:200709982* 2020.

3. Aiolli F, Donini M. EasyMKL: a scalable multiple kernel learning algorithm. *Neurocomputing* 2015; **169:** 215-224.
